# A Case-Control Study of the Correlation Between Blood Parameters and Obesity

**DOI:** 10.7759/cureus.69809

**Published:** 2024-09-20

**Authors:** Ahmet Aydin, Sabin Goktas Aydin

**Affiliations:** 1 Internal Medicine, Medipol University Hospital, Istanbul, TUR; 2 Medical Oncology, Kanuni Sultan Suleyman Training and Research Hospital, Istanbul, TUR

**Keywords:** body mass index: bmi, community obesity, obesity and overweight, obesity prevalence, waist-hip ratio

## Abstract

Background: Obesity has become a global health crisis in adults, and is linked to conditions like diabetes, cardiovascular diseases, and cancer. This study explored associations between body mass index (BMI) and laboratory parameters in healthy individuals to identify risk factors and guide targeted interventions in Turkey. It was found that screening and lifestyle changes can help prevent and manage obesity-related health issues.

Methods: This retrospective case-control study analyzed 2153 adult participants using medical records between 2021 and 2024. The study included those with good overall health; those under 18 years of age or had organ failure, chronic metabolic disorders, obesity complications, or were on multiple obesity-related medications were excluded. Data collected included demographic details, waist-to-hip ratio (WHR), BMI, and laboratory findings. Statistical analyses, including Pearson and Spearman correlations, Mann-Whitney U test and t test, and receiver operating characteristic analysis, were performed using SPSS 24.0 (IBM Corp., Armonk, NY).

Results: The study, comprising 1016 men and 1137 women, revealed that 31.8% of adults were obese. Gender disparities were evident, with a higher prevalence of obesity observed in women: 76.5%, 68.8%, and 45.3% for classes 1, 2, and 3, respectively, compared to corresponding rates of 23.5%, 31.2%, and 54.7% in men. BMI significantly correlated with WHR. Despite the disparity between BMI and WHR between men and women, positive correlations were found between BMI and age (r=0.4) and serum uric acid (SUA) levels (r=0.5). The Mann-Whitney U test also demonstrated a significant association between BMI and fasting plasma glucose level, low-density lipoprotein (LDL), triglycerides, alanine aminotransferase (ALT), uric acid, platelet count, and lymphocyte count (all p values<0.005). Despite the poor correlation with BMI, SUA levels emerged as a potential obesity predictor, with a 4.1 mg/dl cutoff value, exhibiting 50% sensitivity and 34% specificity (p<0.001; area under the curve, or AUC, 0.67; 95% CI 0.65-0.70). There was no significant link between BMI and aspartate aminotransferase, hemoglobin, mean platelet volume, neutrophil and lymphocyte count, vitamin D, thyroid-stimulating hormone, and free thyroxine 4 levels.

Conclusion: This study found significant associations between BMI and laboratory parameters, including serum uric acid, fasting glucose, LDL, triglycerides, and ALT. WHR was also closely linked to BMI, with notable gender differences in body composition. These significant findings underscore the complex nature of obesity and highlight the importance of gender-specific considerations and biomarkers in research and management strategies that are crucial for understanding and addressing this global health crisis.

## Introduction

In recent decades, obesity has emerged as a global health crisis, with associated conditions varying by region. According to meta-analyses and reports, the prevalence of obesity in Turkey is 28.5% [1]. Obesity, characterized by excessive body fat, is primarily classified by body mass index (BMI), though this is a limited measure [2]. It often leads to a range of other health issues, including type 2 diabetes, cardiovascular diseases, and certain cancers [3]. Multifactorial polygenic obesity, influenced by genetic and environmental factors like diet and lack of exercise, requires a thorough understanding of its various phenotypes in adults [4].

Screening for overweight and obesity can significantly alter patient care in three key ways. Firstly, for obese adults with obesity-related diseases, even modest weight loss (5%-10% of the total body weight) can lead to better management of these conditions. Secondly, in obese adults without obesity-related diseases, lifestyle interventions like initiating an exercise regimen can lower the risk of developing conditions. Lastly, for overweight adults who are otherwise healthy, advocating for healthy lifestyle practices can serve as a preventive measure against future obesity [5].

In addition, obesity incurs significant costs. For instance, compared to a metabolically healthy 20-year-old with average weight, having obesity results in lifetime third-party payer costs averaging $14,059, productivity losses of $14,141, and total societal costs of $28,020. Meanwhile, being overweight incurs $5,055 in third-party payer costs, $5,358 in productivity losses, and total societal costs of $10,365. For a metabolically healthy 50-year-old, being obese adds $15,925, $20,120, and $36,278 in costs, whereas being overweight adds $5,866, $10,205, and $16,169 in total, respectively [6]. These substantial economic burdens underscore the importance of effective weight management and prevention strategies.

Many studies examined the association between obesity and lipid profile [7], serum uric acid (SUA) [8], kidney functions [9], liver functions and fatty liver disease [10], and thyroid functions [11]. Some results were sustained; however, some, such as vitamin D deficiency, were controversial [12,13]. A recent marker, SUA, is worth discussing for its implications in metabolic health. Elevated SUA levels independently predict obesity, metabolic syndrome, nonalcoholic fatty liver disease, and diabetes. Uric acid contributes to these conditions through oxidative stress and endothelial dysfunction, exacerbating insulin resistance and inflammation in adipose and liver tissues [8].

BMI and waist-to-hip ratio (WHR) are anthropometric indicators of body composition. Although WHR is strongly correlated with central obesity, it was shown to be more specific for predicting disorders such as diabetes and heart disease [14-16]. BMI is still commonly used to define and classify obesity [17].

The primary objective of this study was to investigate the associations between BMI and various laboratory parameters in healthy individuals. By elucidating the links between BMI and these laboratory markers, we aimed to identify potential risk factors associated with obesity. The secondary objectives of the study were to evaluate the obesity rate in Turkey and identify key laboratory parameters for the assessment of obese individuals. This could inform targeted screening strategies and interventions to mitigate the adverse outcomes associated with aberrations in these laboratory findings through weight management interventions.

## Materials and methods

This retrospective case-control study was conducted by reviewing medical records from an internal medicine outpatient clinic between 2021 and 2024. The sample size for this study was determined to ensure adequate statistical power to detect significant associations between BMI and laboratory parameters. Based on the effect size of d = 0.5, a power level of 80%, and a significance level of 5%, the minimum required sample size was calculated to be 64 participants per group. A total of 3000 medical records were screened, and 2153 participants, aged over 18, were included based on their overall health. Exclusion criteria were organ failure, known chronic metabolic disorders, complications from obesity, and the use of multiple drugs that cause or treat obesity.

Participants were categorized into case and control groups based on their BMI. The control group, including participants with a BMI classified as average weight (BMI 18.5-24.99), was used for comparative analysis with the case group, which included participants with a BMI classified as overweight (BMI ≥25.0) or obese (BMI ≥30.0). The case group was further divided into sub-groups based on the severity of obesity (classes 1, 2, and 3) [18].

Medical records were systematically reviewed to gather comprehensive demographic and clinical data. These included age, gender, waist circumference (measured at the narrowest point between the ribs and iliac crest), hip circumference (measured at the widest point over the buttocks), and laboratory findings. All laboratory tests were performed using standardized instruments. The laboratory parameters examined included renal function tests (serum creatinine, blood urea nitrogen, estimated glomerular filtration rate), liver function tests (alanine aminotransferase, or ALT; aspartate aminotransferase, or AST; gamma-glutamyl transferase), thyroid function tests (thyroid-stimulating hormone, or TSH; free thyroxine 4, or T4), vitamin D levels, SUA, and lipid parameters (low-density lipoprotein cholesterol, or LDL-C; high-density lipoprotein cholesterol, or HDL-C; triglycerides).

WHR is calculated by dividing waist circumference by hip circumference. For women, a WHR of 0.85 or less, and for men, 0.9 or less is considered the cutoff point. BMI, calculated as weight in kilograms divided by the square of height in meters, BMI = weight (kg)/height (m^2^), falls into five categories: underweight (BMI <18.5), average weight (BMI 18.5-24.99), overweight (BMI 25.0-29.9), class 1 obesity (BMI 30.0-34.9), class 2 obesity (BMI 35.0-39.9), and class 3 obesity (BMI ≥40.0).

All patients or their families were fully informed about the study's purpose and requirements, and informed written consent was obtained from all participants. The Local Ethics Committee of Istanbul Medipol University approved the study in June 2024 (decision number E-10840098-772.02-3143).

Statistical analysis

Statistical analyses were performed using SPSS 24.0 software (IBM Corp., Armonk, NY). The distribution of data was assessed using the Shapiro-Wilk test. The Pearson correlation was applied to variables with a normal distribution, while the Spearman correlation was used for those without a normal distribution. Descriptive statistics for non-normally distributed parameters were presented as median values. The Mann-Whitney U test and two-sample t-test were used to assess the association between BMI groups and laboratory findings. Additionally, receiver operating characteristic (ROC) analyses were conducted to determine cut-off values for laboratory findings potentially correlated with BMI. All statistical tests were two-sided; p-values less than 0.05 were considered statistically significant.

## Results

**Figure 1:**
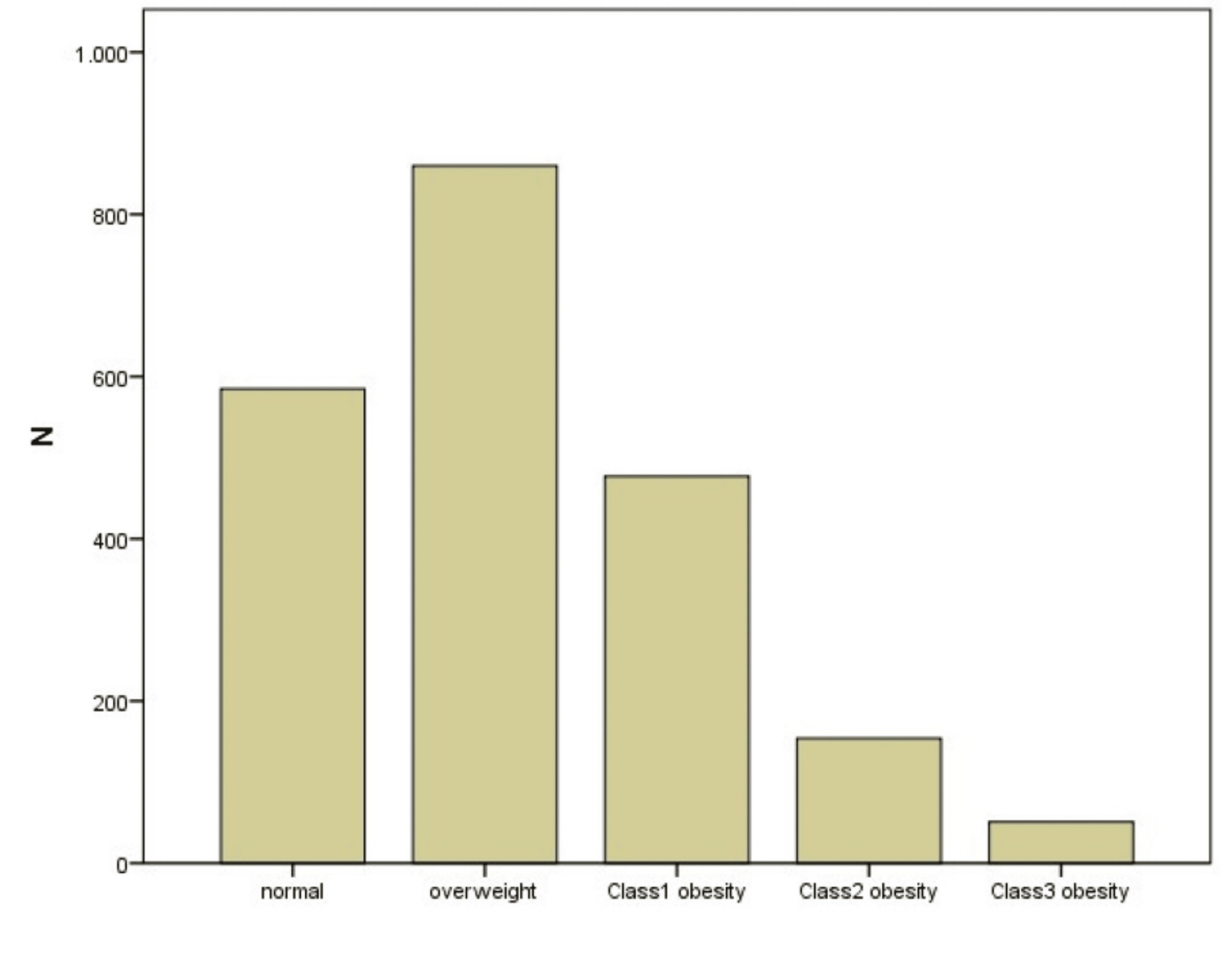
Number of participants across the BMI groups BMI, body mass index

This study comprised 2153 participants, with 1016 (47.2%) men and 1137 (52.8%) women. Their median age was 45 years (range: 18-84). Among the participants, 477 (22.2%) were classified as having class 1 obesity, 154 (7.2%) as class 2, and 51 (2.4%) as class 3 obesity. Additionally, 860 individuals (39.9%) were overweight, while only 611 (28.4%) fell within the normal BMI range (Figure 1).

**Figure 2:**
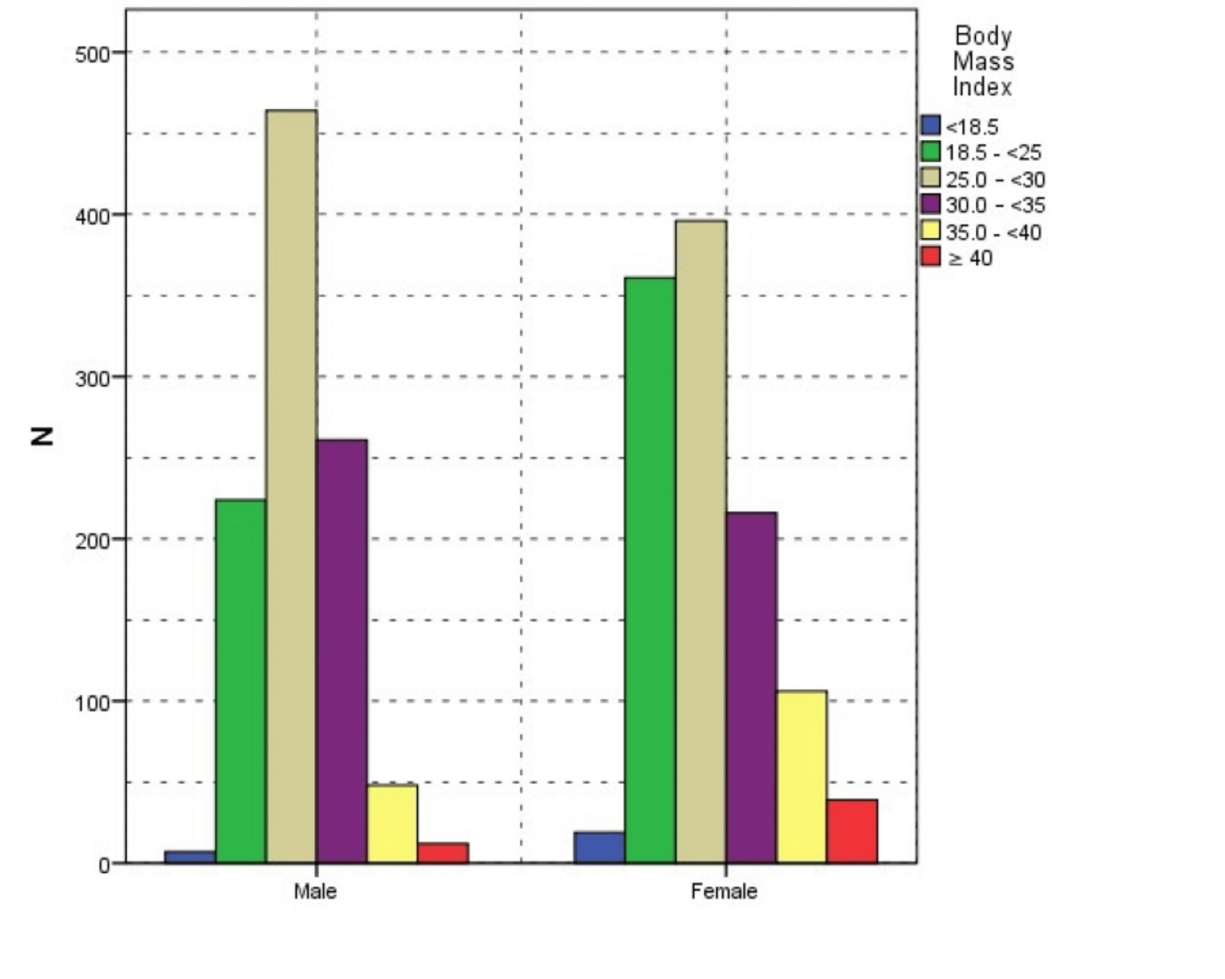
Body mass index across genders

The classification of obesity revealed that among female participants, 39 (76.5%), 106 (68.8%), and 216 (45.3%) fell into class 1, class 2, and class 3 categories, respectively. In contrast, 12 (23.5%), 48 (31.2%), and 261 (54.7%) male participants fell in the corresponding obesity classes (Figure 2). Furthermore, women displayed a significantly higher BMI than men (p<0.001).

**Figure 3:**
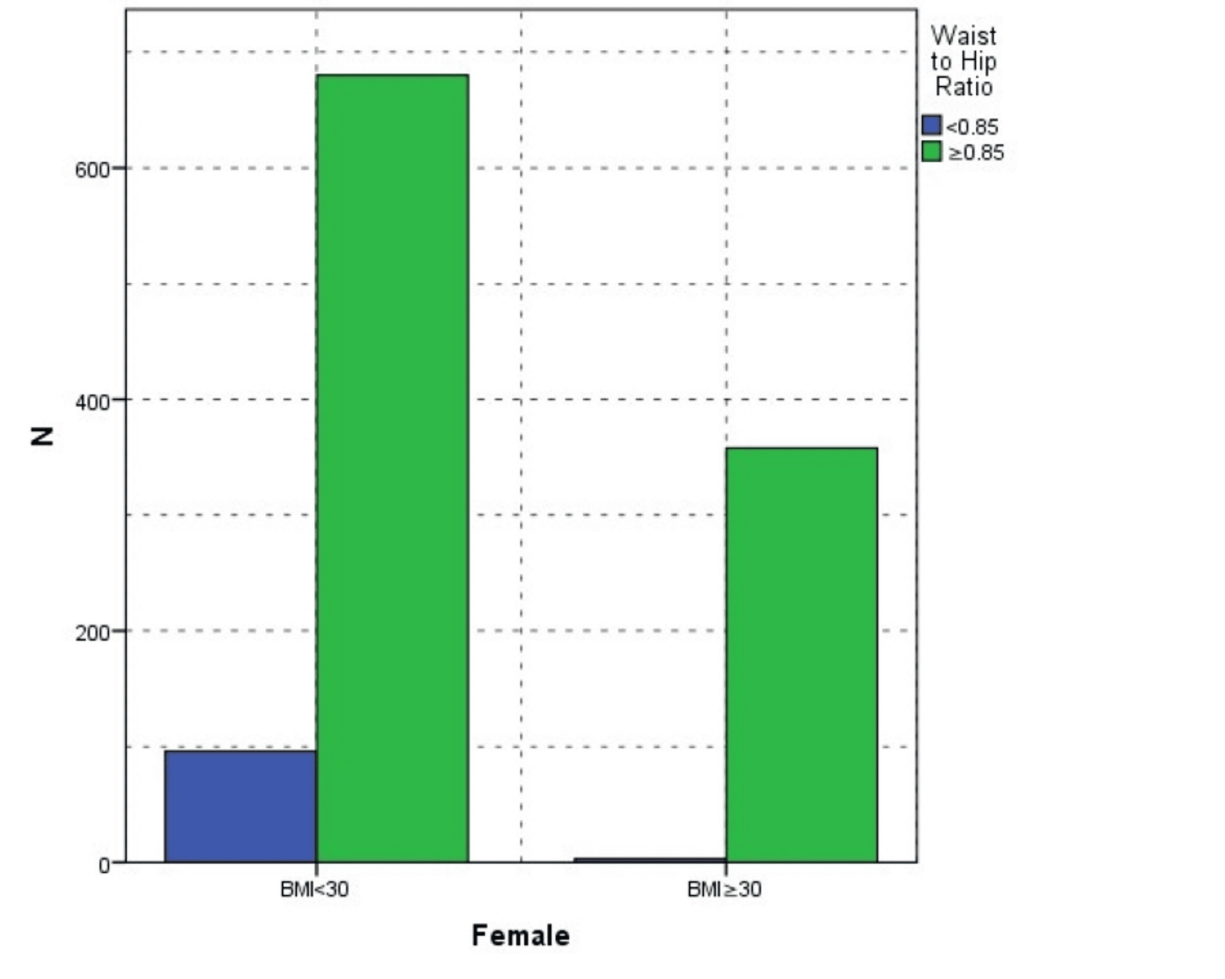
Association between obesity and waist-to-hip ratio in female patients BMI, body mass index

**Figure 4:**
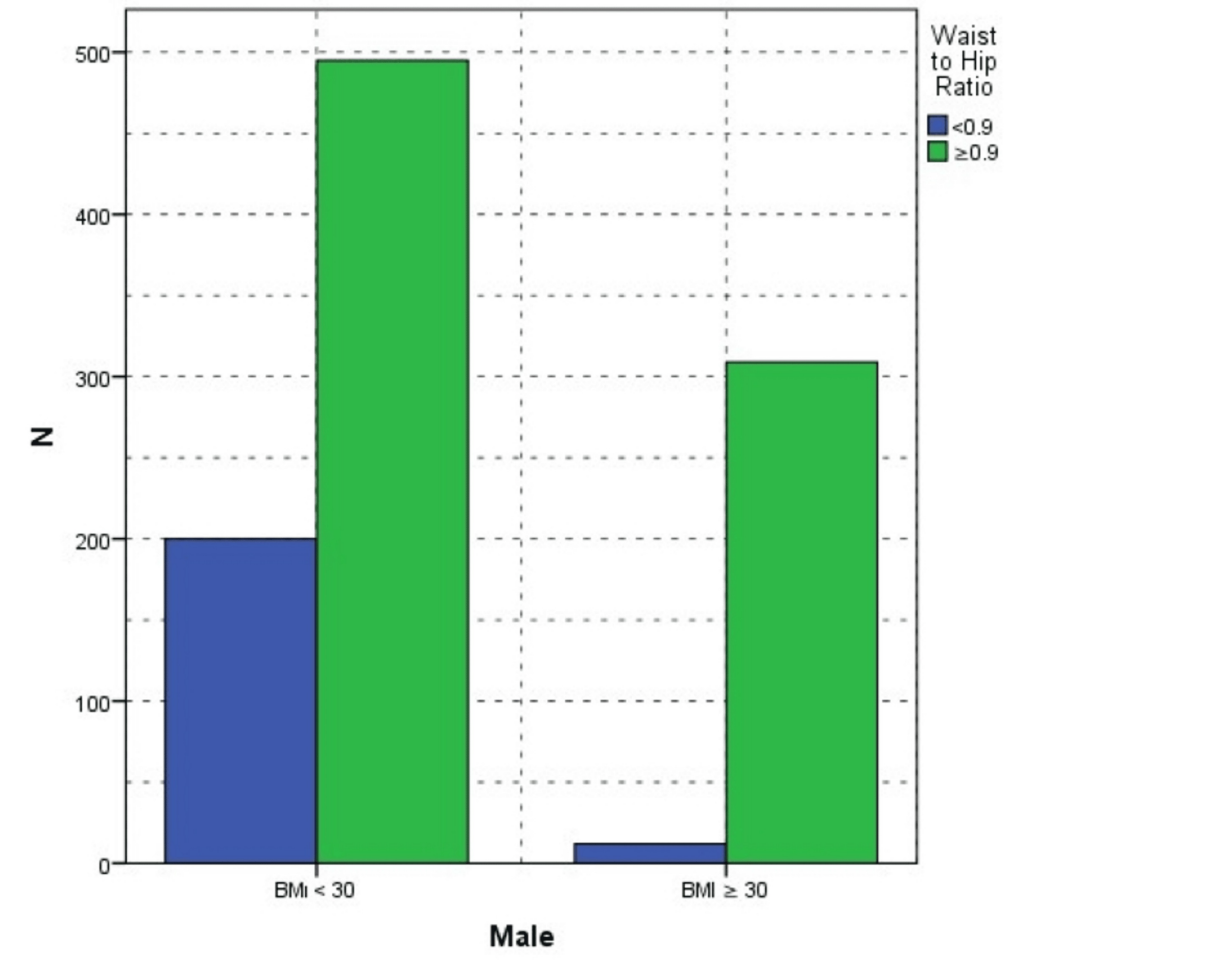
Association between obesity and waist-to-hip ratio in male patients BMI, body mass index

Aging significantly affected the WHR (r=0.3, p<0.001); WHR was significantly correlated with BMI (r=0.5, p<0.01). Compared to gender, an increased WHR was noted in 37.3% of men and 48.2% of women, also showing statistical significance (p<0.001). On the other hand, 12 (5.7%) men with a BMI ≥25 had a normal WHR and 95 (11.8%) men with a BMI <25 had an increased WHR. Only three women with a BMI ≥25 had a normal WHR. However, 294 (28.3%) women had an increased WHR in the normal BMI group (Figures 3-4).

**Figure 5:**
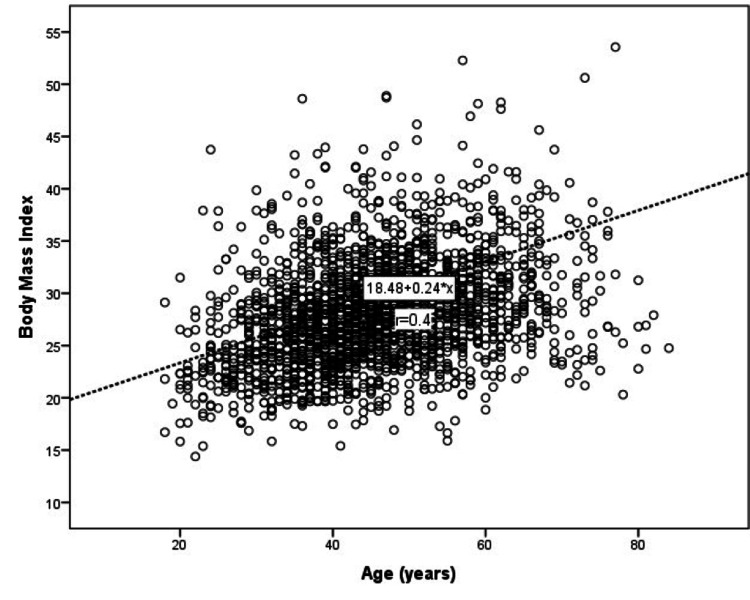
Correlation between age and body mass index x is the independent variable, and r is the correlation coefficient.

In the overall cohort, a significant and moderate positive correlation existed between BMI and age (r=0.4) (Figure 3) and SUA (r=0.5) (all p-values <0.05) (Figure 5). Table 1 summarizes means ± SDs for age in relation to the BMI groups.

**Table 1:** Means ± SDs for age in relation to the body mass index

Body mass index	Age (mean ± SD)	p-value
18.5 to <25	40.4 ± 11.7	<0.05
25.0 to <30	45.9 ± 10.6	
30.0 to <35	49.2 ± 9.9	
35.0 to <40	50.9 ± 12.1	
≥40	51.9 ± 11.3	

**Figure 6:**
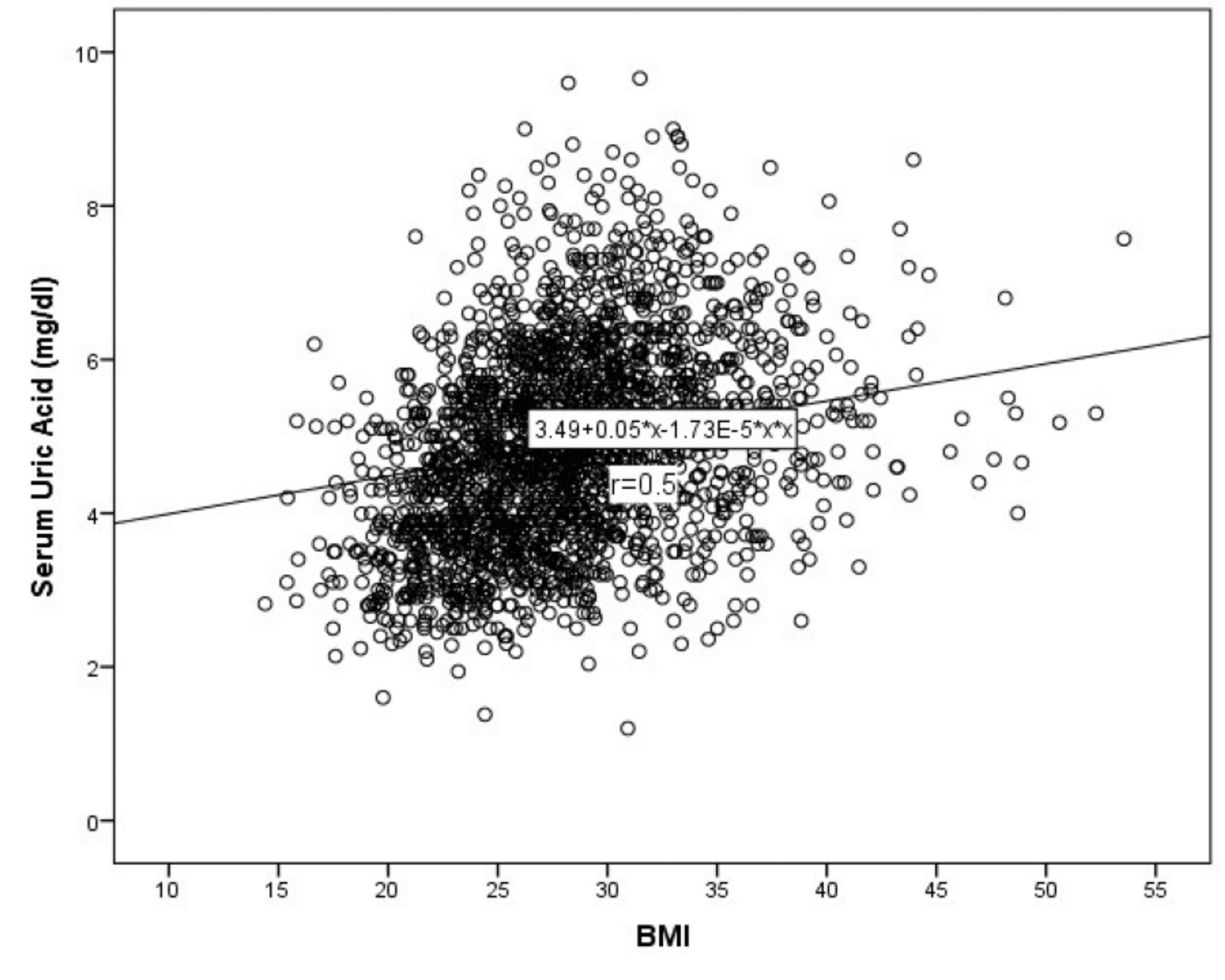
Correlation between the serum uric acid level and body mass index (BMI) x is the independent variable, and r is the correlation coefficient.

When comparing participants with and without obesity, the study demonstrated that BMI was significantly linked with fasting plasma glucose level, LDL, triglycerides, ALT, uric acid (Table 2, Figure 6), platelet count, and lymphocyte count (all p-values <0.05. However, there was no significant association with TSH, hemoglobin, AST, creatinine, neutrophil count, mean platelet volume (MPV), HDL, or vitamin D levels.

**Table 2:** Comparison of the median serum uric acid level in relation to the body mass index

Body mass index	Serum uric acid (mg/dl), median (range)	p-value
18.5 to <25	4.09 (1.38-8.40)	<0.05
25.0 to <30	4.90 (2.04-9.60)	
30.0 to <35	5.43 (1.20-9.66)	
35.0 to <40	5.40 (2.50-8.50)	
≥40	5.44 (3.30-8.60)	

As expected, a significant association was found between BMI, WHR, and body fat percentage (all p-values <0.001). When the data was stratified according to gender, a significant association between BMI and age, ALT, SUA, fasting blood glucose, and triglycerides was found in men. However, women's significant links were age, HDL, LDL, triglyceride level, platelet count, SUA, ALT, and fasting blood glucose (all p-values <0.05). Neither the total cohort nor different genders displayed any correlation between BMI and AST, hemoglobin, MPV, neutrophil and lymphocyte count, vitamin D, TSH, and free T4 levels (Table 3).

**Table 3:** Mean ± SD values for the laboratory parameters, comparing obese and non-obese patients ALT, alanine aminotransferase; AST, aspartate aminotransferase; FBC, fasting blood glucose; HDL, high-density lipoprotein; LDL, low-density lipoprotein; TSH, thyroid-stimulating hormone; BMI, body mass index

	Male BMI <30	Male BMI ≥30	p-value	Female BMI <30	Female BMI ≥30	p-value
ALT (U/l)	26.1 ± 15.7	23.5 ±9.2	<0.05	16.3 ± 8.2	18.7 ± 6.3	<0.05
AST (U/l)	22.8 ± 6.2	23.5 ± 9.2	>0.05	18.6 ± 5.6	18.9 ± 4.2	>0.05
FBC (mg/dl)	97.3 ± 10.9	108.6 ± 21.7	<0.05	92.4 ± 9.6	104.0 ± 19.1	<0.05
HDL (mg/dl)	44.5 ± 11.2	44.2 ± 10.7	>0.05	58.5 ± 13.0	55.9 ± 14.5	<0.05
LDL (mg/dl)	117.8 ± 32.4	131.4 ± 33.5	>0.05	124.7 ± 38.9	133.2 ± 31.7	<0.05
Triglycerides (mg/dl)	152.0 ± 98.4	163.0 ± 78.0	<0.05	103.4 ± 58.3	123.5 ± 62.6	<0.05
Platelet (10^3^/ml)	238.7 ± 46.5	248.9 ±54.5	>0.05	268.8 ± 63.2	290.2 ± 65.3	<0.05
Hemoglobin (g/dl)	14.0 ± 1.1	15.0 ± 1.1	>0.05	12.9 ± 1.1	12.6 ± 1.4	>0.05
Neutrophil (10^3^/ml)	3.9 ± 1.3	3.9 ± 1.4	>0.05	3.7 ± 1.3	4.0 ± 1.4	>0.05
Lymphocyte (10^3^/ml)	2.4 ± 0.6	2.6 ± 0.7	>0.05	2.2 ± 0.7	2.4 ± 0.6	>0.05
Vitamin D (ng/ml)	21.1 ± 12.9	18.7 ± 9.6	>0.05	20.0 ± 12.8	17.3 ± 12.5	>0.05
TSH (mIU/l)	2.4 ± 2.2	2.6 ± 2.4	>0.05	2.3 ± 2.0	2.6 ± 1.5	>0.05

**Figure 7:**
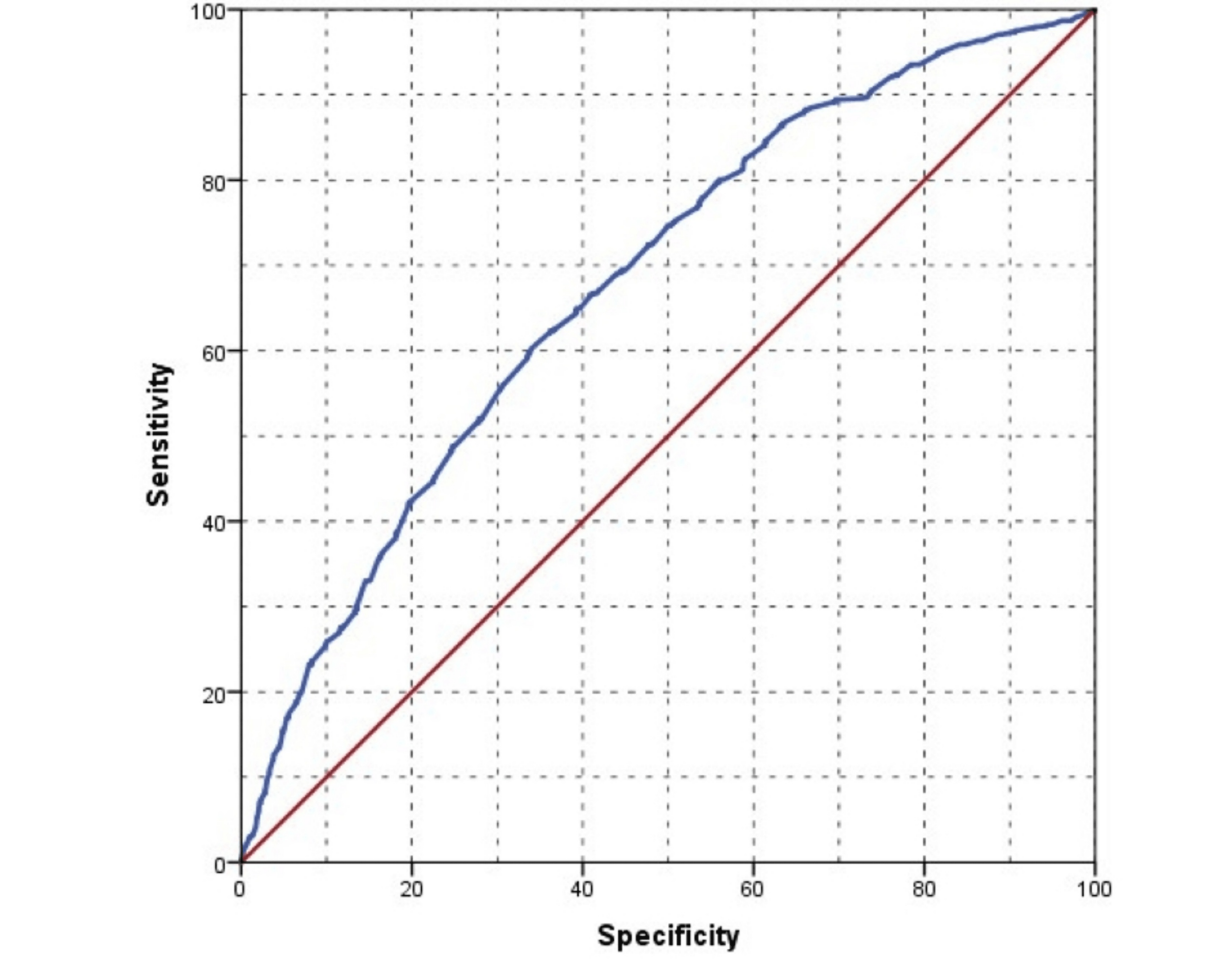
Receiver operating characteristic (ROC) analysis assessing the predictive value of serum uric acid levels for obesity

In our study correlating blood parameters with BMI, numerous factors showed significant associations, yet only SUA levels emerged with a discernible cut-off value through ROC analysis. Specifically, at a 4.1 mg/dl threshold, serum uric acid exhibited 50.0% sensitivity and 34.0% specificity, marking its significance in obesity among healthy adults (Figure 7).

## Discussion

This study delved into the global obesity crisis, particularly prevalent in Turkey, affecting 28.5% of the population. Beyond its classification by BMI, obesity encompasses a spectrum of health issues linked to genetic and environmental factors. We investigated associations between BMI and various laboratory parameters in healthy individuals, aiming to identify risk factors associated with obesity. Through this exploration, we sought to inform targeted screening strategies and interventions to mitigate adverse outcomes.

Obesity is more common in women than men, which can be explained by behavioral and food differences between genders or differences in the neural food response in the brain [19,20]. A recent review also indicated that obesity, a multifactorial condition with complex interactions, tends to be higher in women [21]. The 2022 Turkiye Health Survey identified an overall obesity rate of 20.2%, with females exhibiting a 23.6% obesity rate and a 30.9% pre-obesity rate, while males had a 16.8% obesity rate and a 40.4% pre-obesity rate [22]. However, in our study, we found that among participants, 22.2% were classified with class 1 obesity, 7.2% with class 2, and 2.4% with class 3. Moreover, 39.9% were overweight, with only 28.4% falling within the normal BMI range. When examining obesity classifications among females, 76.5%, 68.8%, and 45.3% were categorized into classes 1, 2, and 3, respectively, while among males, the corresponding percentages were 23.5%, 31.2%, and 54.7%, respectively. Additionally, women displayed a notably higher BMI than men (p<0.001).

Changes in metabolic control, insulin insensitivity, inflammation, and weakened immune response are key features shared by obesity and aging. The fat tissue in obese individuals displays signs of aging, such as faulty mitochondria and cellular dysfunction. The metabolic irregularities linked with obesity resemble those seen in typical aging, and there is significant evidence pointing to obesity's potential to hasten aging processes [23]. Our study has confirmed previous study findings [1,23] by showing that aging was significantly correlated with obesity rates. However, sarcopenic obesity, a pressing issue, was not assessed in our study, which was one of the limitations.

Many recent studies have demonstrated that WHR is more reliable for understanding obesity and its related complications, including mortality [13,15,24]. On one hand, Widjaja et al. studied 208 obese adolescents, half of whom had metabolic syndrome. They found a significant link between WHR rather than BMI and metabolic syndrome in obese teens [16]. On the other hand, Zhu et al. argued that relying solely on BMI and WHR for obesity classification can lead to misdiagnosis. They discovered that Chinese military personnel had remarkably low obesity rates according to BMI (0.6%) and body fat percentage (0.3%). When overweight individuals were included, the prevalence of overweight/obesity determined by BMI and WHR was higher than that based on body fat percentage. Compared to body fat percentage, BMI and WHR had a higher rate of false positives when identifying obesity [25]. In our study, 5.7% of men with a BMI ≥25 had a normal WHR, while 11.8% of men with a BMI <25 exhibited an increased WHR. Conversely, only three women with a BMI ≥25 displayed a normal WHR, while 28.3% of women in the normal BMI group had an increased WHR. The disparity between BMI and WHR was evident between men and women, highlighting distinct body composition patterns across genders. Another limitation of the study was that it did not analyze participants' body fat percentage.

Vitamin D adequacy has been shown to have an inverse association with the prevalence of obesity [14]. However, our study did not demonstrate a significant link between BMI and vitamin D levels.

A review of the hematological consequences of obesity indicated that, compared with non-obese controls, obese people had significantly elevated white blood cell count and platelet count, and lower hemoglobin levels [26]. Dyslipidemia is often present in obese people, and many studies showed that obese people had increased LDL and decreased HDL levels [27]. Another well-known laboratory finding was altered liver function tests, which were significantly related to non-alcoholic fatty liver disease in obese individuals [28]. Meta-analyses have also proven an association between serum uric acid levels and metabolic syndrome [29]. Xu et al. showed that elevated serum uric acid levels were associated with a high body mass index, hypertension, and hyperglycemia [30]. A complex and mutual relationship between the thyroid axis and obesity has been established by Walczak et al. They showed that subclinical hypothyroidism may induce changes in the basal metabolic rate with subsequent increases in the BMI, but obesity can also affect thyroid function via several mechanisms [11].

Our study confirmed several established associations between obesity and laboratory markers. Similar to prior researches [26, 27], we observed that an increased BMI linked to elevated white blood cells, platelets, LDL-C, and uric acid, and decreased HDL-C. Additionally, we found significant correlations between BMI and markers of liver health (ALT) and blood sugar (fasting glucose) in both men and women. However, some of our findings differed slightly from previous studies. While others reported lower hemoglobin in obese individuals [26], we did not observe this association.

We found no significant link between BMI and TSH levels. In contrast to our findings, where no significant relationship was observed between TSH levels and BMI, Xu et al., reported a significant association [30]. Additionally, the study by Mele et al. identified a significant association between TSH and BMI in a large cohort of euthyroid individuals with obesity. Their results indicated an upward trend in TSH levels in females with increasing BMI, while TSH levels also correlated with body composition parameters such as fat mass and fat-free mass [31]. These differences may be attributable to the larger sample size in their cohort, gender-specific metabolic factors, or the influence of smoking, which we did not assess in our study.

Limitations

This study also had several limitations. The retrospective design limits causal inferences between obesity and laboratory markers, as it relies on historical data without the ability to control for temporal changes or interventions. BMI used as the primary measure of obesity may not fully capture body composition, especially the distinction between fat and muscle mass. The exclusion of body fat percentage analysis further limits the findings. Additionally, the study population data, drawn from an outpatient clinic, may not represent the general population, introducing potential selection bias. Lastly, unaccounted factors like diet, physical activity, and genetics may have influenced the observed associations. Future research should address these aspects for a more comprehensive understanding.

## Conclusions

In conclusion, this study found significant associations between BMI and laboratory parameters, including serum uric acid, fasting glucose, LDL, triglycerides, and ALT. Moreover, our single-center study showed that 31.8% of adults were obese. Age and gender were significantly linked to obesity. The serum uric acid level can be used as a reliable predictor of obesity with a 4.1 mg/dl threshold. It could serve as a valuable biomarker for assessing the risk of developing obesity. Gender-based analyses revealed some interesting distinctions. Men with a higher BMI had significant associations with ALT and SUA, whereas women displayed a stronger connection with HDL and LDL cholesterol. WHR was also closely linked to BMI, with notable gender differences in body compositions.

These significant findings underscore the complex nature of obesity and highlight the importance of gender-specific considerations and biomarkers in research and management strategies that are crucial for understanding and addressing this global health crisis. Additionally, understanding these associations can have substantial implications for cost outcomes, as obesity-related conditions are known to incur significant costs. By improving targeted screening and intervention strategies, healthcare systems can potentially reduce the financial burden associated with obesity, thereby aligning better with cost-effective healthcare management.
